# Multisystem laboratory changes before and after blood purification in acute pancreatitis: a real-world descriptive analysis of 141 cases

**DOI:** 10.3389/fmed.2026.1850265

**Published:** 2026-09-23

**Authors:** Guoying Ma, Cendan Lu, Honglin Xiang, Mingyan Wu, Xiaoqin Tan, Jing Wang

**Affiliations:** Department of Nephrology, The People's Hospital of The Qiandongnan Miao and Dong Autonomous Prefecture, Kaili, Guizhou, China

**Keywords:** acute pancreatitis, blood purification, laboratory trajectories, multi-system profiling, real-world evidence

## Abstract

**Background:**

Acute pancreatitis (AP) is a dynamic, multisystem disorder. Blood purification (BP) is increasingly utilized in severe or hypertriglyceridemia-associated AP, yet the overall patterns and cross-dimensional heterogeneity of post-BP laboratory shifts remain poorly characterized.

**Objective:**

To map the patterns, magnitudes, cross-system correlations, and patient-level heterogeneity of paired laboratory parameter changes observed before and after BP in AP patients using real-world data, strictly without inferring causal efficacy.

**Methods:**

This retrospective observational study analyzed 141 consecutive AP patients receiving BP (hemoperfusion, plasma exchange, or combined). Paired laboratory indicators across seven biological domains were compared. An exploratory multisystem laboratory change score (0∼6) was constructed to evaluate directional consistency. Rule-based stratification was applied to examine change patterns alongside change-magnitude ranking and subgroup analyses.

**Results:**

Most laboratory indicators exhibited post-treatment reductions. The largest relative median decreases occurred in IL-6 (−86.79%), serum amylase (−86.67%), and triglycerides (−86.23%). The mean multisystem change score was 4.24 ± 1.34; concurrent directional shifts occurred across ≥3 domains in 87.94% of patients and ≥4 domains in 70.92%. Exploratory stratification identified subgroups exhibiting greater, intermediate, or smaller multidimensional shifts. Correlation analysis demonstrated strong intra-domain synchrony between renal parameters (*r* = 0.726), but weak or non-significant cross-domain correlations.

**Conclusions:**

Patients with AP showed marked, heterogeneous pre- to post-BP multidimensional laboratory shifts. Lacking a contemporaneous control group, these observational changes cannot be causally attributed to BP and reflect the combined influence of natural disease evolution, fluid resuscitation, and concurrent supportive care.

## 1 Introduction

Acute pancreatitis (AP) is a common acute abdominal condition characterized by substantial variability in clinical severity and disease trajectory. The revised Atlanta Classification (2012) categorizes AP into mild, moderately severe, and severe forms, emphasizing that persistent organ failure is the key determinant of severity. This underscores that clinical assessment should not be limited to local pancreatic injury but must focus on multisystem damage and its dynamic evolution (1). At the management level, international guidelines (e.g., IAP/APA and ACG) establish that standard care remains rooted in etiological management, fluid resuscitation, organ support, and complication management (2, 3).

Accumulating evidence demonstrates that AP is a complex, dynamic multisystem process accompanied by lipid metabolic dysregulation, inflammatory cascade amplification, fluctuating pancreatic enzyme activity, and hepatorenal organ dysfunction (1, 4–6). Consequently, laboratory monitoring in AP is shifting from single-biomarker evaluations toward integrated, multidimensional profilingencompassing inflammatory markers (CRP, PCT, IL-6), lipid panels, electrolytes, liver/kidney function, and coagulation parameters to better capture overall disease progression (4–6).

In severe or hypertriglyceridemia-associated AP (HTG-AP), blood purification (BP) techniques (including hemoperfusion, plasma exchange, and continuous renal replacement therapy) are frequently utilized in real-world practice to rapidly lower triglyceride levels and clear circulating inflammatory mediators (7–10). While previous studies suggest temporal shifts in biological markers following BP, existing literature is constrained by small sample sizes, heterogeneous treatment protocols, isolated endpoint reporting, and a lack of real-world multidimensional data integration (7–10). Crucially, in clinical practice, multiple laboratory parameters fluctuate simultaneously, yet whether these changes exhibit consistent cross-dimensional patterns across lipid metabolism, inflammation, organ function, and coagulation remains poorly characterized (5, 6, 11).

Addressing how multidimensional laboratory trajectories evolve in real-world scenarios holds clinical descriptive value (10–12). However, rigorous evaluation of these trajectories requires acknowledging inherent design boundaries. Therefore, this retrospective study analyzes paired, multidimensional laboratory data from 141 consecutive AP patients who underwent BP at a single center. The primary objective is strictly descriptive: to map the patterns, magnitudes, and cross-dimensional heterogeneity of laboratory parameter changes observed before and after BP. Importantly, because all enrolled patients received BP without a parallel non-BP control group, this study is explicitly not designed to establish causal inferencing, attribute observed lab changes solely to BP, or demonstrate therapeutic efficacy.

## 2 Methods

### 2.1 Study population

This was a single-center, retrospective, single-arm descriptive observational study. The design, implementation, and reporting adhered to the Strengthening the Reporting of Observational Studies in Epidemiology (STROBE) statement. Patients with AP who received blood purification (BP) treatment during hospitalization at Qiandongnan Miao and Dong Autonomous Prefecture People's Hospital from January 2020 to December 2025 were continuously screened. The study protocol was reviewed and approved by the Ethics Committee of Qiandongnan Miao and Dong Autonomous Prefecture People's Hospital (Approval No. 2026ER-003). Due to the retrospective observational design and the use of fully de-identified clinical data, the requirement for written informed consent was waived by the Ethics Committee.

A total of 141 patients were included, comprising 97 males and 44 females, with a mean age of 47.01 ± 14.53 years. The cohort was clinically enriched for hypertriglyceridemia-associated and moderate-to-severe acute pancreatitis, reflecting the population in whom BP was preferentially used at our center. Etiology, severity according to the revised Atlanta classification, organ failure, ICU admission, indications for BP, timing and number of BP sessions, and major concomitant treatments were additionally extracted from the medical records and are summarized in Table 1. According to the BP modality, 81 patients received hemoperfusion alone, 45 received single plasma exchange alone, and 15 received combined therapy.

**Table 1:** Clinical characteristics, acute pancreatitis severity, and blood purification characteristics of the study population (*n* = 141).

Characteristic	Value
Demographic characteristics	
Number of patients	141
Age, years, mean ± SD	47.01 ± 14.53
Male, *n* (%)	97 (68.79)
Female, *n* (%)	44 (31.21)
Etiology of acute pancreatitis	
Hypertriglyceridemia-associated AP	96 (68.09)
Biliary AP	27 (19.15)
Alcohol-associated AP	10 (7.09)
Other/idiopathic causes	8 (5.67)
Revised Atlanta classification	
Mild AP	14 (9.93)
Moderately severe AP	55 (39.01)
Severe AP	72 (51.06)
Organ failure present, *n* (%)	79 (56.03)
Persistent organ failure (>48 h), *n* (%)	61 (43.26)
ICU admission, *n* (%)	65 (46.10)
Principal indication for BP	
Severe hypertriglyceridemia	91 (64.54)
Organ dysfunction/high inflammatory burden	32 (22.70)
Combined indications	18 (12.77)
Timing of BP initiation	
≤24 h after admission/diagnosis	83 (58.87)
>24∼48 h	39 (27.66)
>48 h	19 (13.48)
Median number of BP sessions, median (IQR)	2 (1∼3)
Patients receiving >1 BP session, *n* (%)	82 (58.16)
Typical duration per BP session, h	4.0 (3.0∼5.0)
BP modality	
Hemoperfusion alone	81 (57.45)
Single plasma exchange alone	45 (31.91)
Double-filtration plasmapheresis + hemoperfusion	11 (7.80)
Single plasma exchange + hemoperfusion	4 (2.84)
Concomitant treatment	
Intravenous fluid resuscitation	141 (100.00)
Nutritional support	132 (93.62)
Glucose/lipid metabolic management	116 (82.27)
Anti-infective treatment	87 (61.70)
Vasopressor support	39 (27.66)
Mechanical ventilation	31 (21.99)

### 2.2 Inclusion and exclusion criteria

Inclusion criteria: (1) Definite clinical diagnosis of acute pancreatitis according to the revised Atlanta classification criteria, i.e., at least 2 of the following 3 items: ① typical persistent upper abdominal pain; ② serum amylase and/or lipase elevated to ≥3 times the upper limit of normal; ③ imaging (CT or ultrasound) findings indicative of acute pancreatitis; (2) Received blood purification treatment during hospitalization, including hemoperfusion, plasma exchange, or combined therapy; (3) Had paired laboratory test data available before and after blood purification that could be used to characterize pre- and post-BP laboratory changes; (4) Clear source of case data allowing completion of basic clinical information extraction and statistical analysis.

Exclusion criteria: (1) Duplicate records during the same hospitalization; (2) Missing key baseline data such as age, sex, or blood purification modality, making it impossible to define case characteristics or treatment type; (3) Severely missing laboratory data before and after treatment, preventing effective paired comparison; (4) Laboratory data with obvious errors, unclear units, or inability to verify; (5) Inability to determine the specific pre- or post-treatment time point corresponding to the test results.

A total of 246 patients with acute pancreatitis who were initially screened for blood purification were assessed for eligibility. After excluding 105 cases due to data recording issues (*n* = 12), insufficient diagnostic evidence (*n* = 8), missing or unpaired laboratory data (*n* = 65), and data quality or traceability problems (*n* = 20), 141 patients were finally enrolled in the study. These patients were classified according to blood purification modality: hemoperfusion alone (*n* = 81), single plasma exchange alone (*n* = 45), and combined therapy [*n* = 15; including double filtration plasmapheresis plus hemoperfusion [*n* = 11] and single plasma exchange plus hemoperfusion [*n* = 4]]. The study selection process and grouping are summarized in Figure 1.

**Figure 1:**
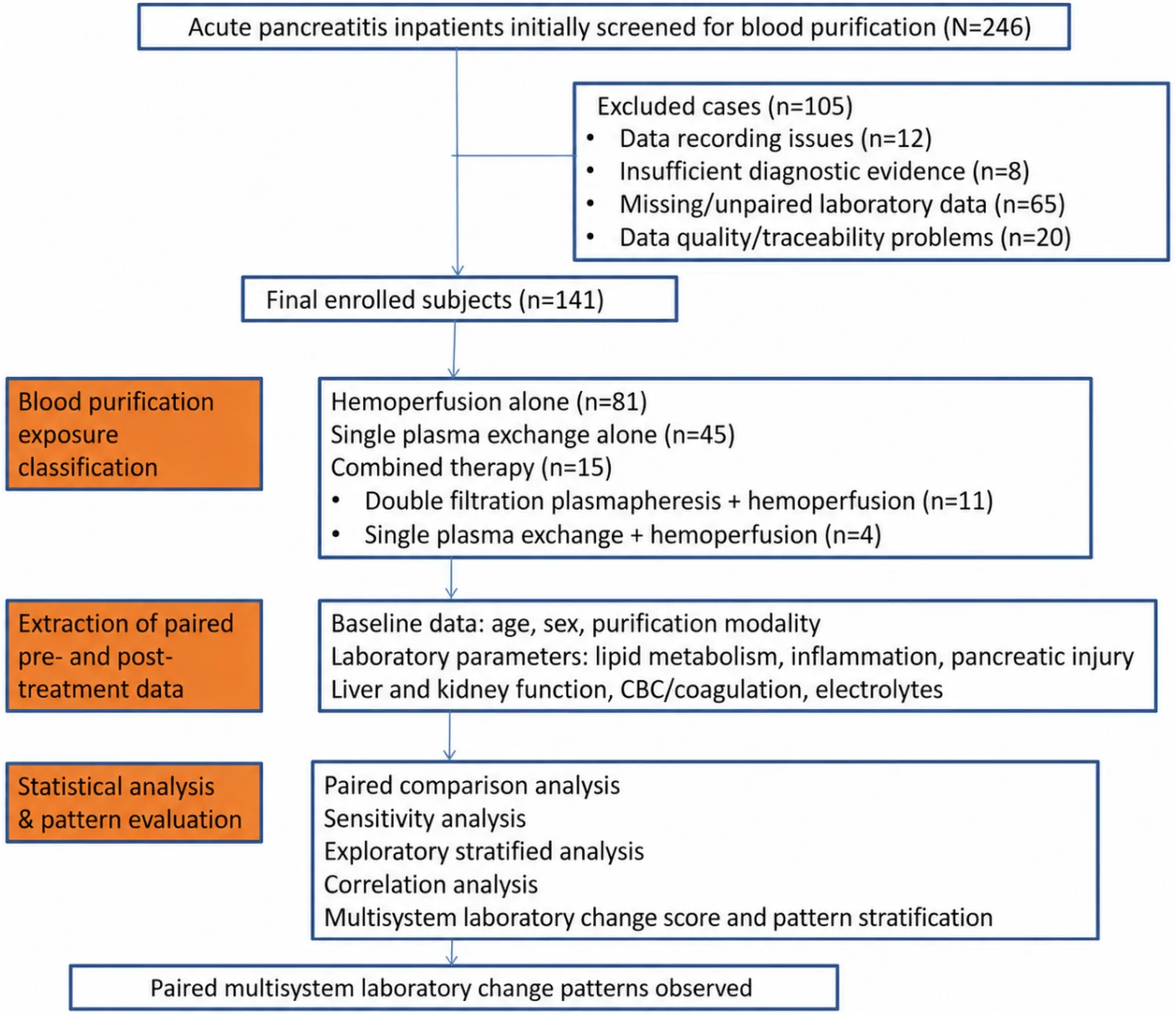
Study flow diagram.

### 2.3 Definition of blood purification exposure

Blood purification exposure was defined according to the actual treatment modality recorded in the original database. Blood purification modalities included hemoperfusion alone, single plasma exchange alone, double filtration plasmapheresis combined with hemoperfusion, and single plasma exchange combined with hemoperfusion. In this study, blood purification was considered as an exposure factor implemented clinically, rather than a randomly assigned intervention. Therefore, the primary analysis was restricted to descriptive within-patient comparisons of laboratory measurements obtained before and after BP. These comparisons were not intended to estimate the causal effect or therapeutic efficacy of BP, either overall or between different BP modalities.

In routine clinical practice, the indication for BP was determined by the treating team according to the overall clinical condition rather than by a standardized study protocol. The principal indications included severe hypertriglyceridemia, marked systemic inflammatory burden, organ dysfunction/failure, or a combination of these features. The timing, number, and duration of BP sessions were individualized according to clinical status and laboratory evolution. Because BP modality and intensity were not randomly assigned, these treatment characteristics were considered descriptive variables and were not interpreted as comparative treatment effects.

### 2.4 Data collection and observed variables

Clinical characteristics and laboratory indicators were extracted from the electronic medical records. In addition to age, sex, and BP modality, clinical variables included AP etiology, disease severity according to the revised Atlanta classification, presence of organ failure, ICU admission, clinical indication for BP, interval from AP diagnosis/admission to BP initiation, number and duration of BP sessions, and major concomitant treatments, including fluid resuscitation, nutritional support, glucose and lipid management, anti-infective therapy when clinically indicated, and organ-support measures. Laboratory indicators included in the analysis covered the following dimensions: (1) Lipid metabolism indicators: total cholesterol, triglycerides; (2) Inflammation-related indicators: C-reactive protein (CRP), interleukin-6 (IL-6), tumor necrosis factor-alpha (TNF-α), procalcitonin (PCT); (3) Pancreatic injury indicator: serum amylase; (4) Renal function indicators: blood urea nitrogen, creatinine; (5) Liver function indicators: bilirubin, alanine aminotransferase, albumin; (6) Complete blood count and coagulation indicators: white blood cell count (WBC), neutrophil percentage (N), prothrombin time (PT); (7) Electrolyte indicators: potassium (K), calcium (Ca).

For each laboratory variable, the pre-BP value was defined as the closest available measurement obtained before initiation of the first BP session, and the post-BP value was defined as the first available measurement obtained after completion of that session. The median interval from the pre-BP blood draw to BP initiation was 2.3 h (IQR 1.1∼4.8 h), and the median interval from completion of BP to the post-BP blood draw was 11.6 h (IQR 6.2∼18.9 h). Overall, 112 of 141 patients (79.43%) had their principal post-BP laboratory assessment performed within 24 h after completion of BP, 21 (14.89%) between >24 and 48 h, and 8 (5.67%) after 48 h. Because sampling schedules were determined by routine clinical care rather than by a prespecified protocol, the exact interval differed across patients and laboratory variables. Accordingly, the primary analyses should be interpreted as descriptive paired comparisons rather than standardized kinetic measurements. If multiple test values existed within the same phase, the values preset for analysis in the original data table were used. Test results that could not be clearly assigned to specific pre- or post-treatment time points were not included in the paired analysis.

### 2.5 Study outcomes

#### 2.5.1 Primary outcome

The primary outcome was the pattern of paired multisystem laboratory changes observed between the pre- and post-BP measurements, including changes in inflammatory markers, lipid metabolism, pancreatic injury markers, organ function, coagulation parameters, and electrolyte balance. This outcome was intended solely to describe the direction and magnitude of laboratory changes over the observed clinical course and was not designed to establish that these changes were attributable to BP.

#### 2.5.2 Secondary outcomes

Secondary outcomes included: (1) Absolute changes in each laboratory indicator; (2) Relative percentage changes from baseline for each laboratory indicator; (3) Exploratory, hypothesis-generating stratified analyses by blood purification modality and baseline biological burden where sample size permitted; (4) Patient-level descriptive multisystem laboratory change score; (5) Descriptive assessment of the consistency of directional changes; and (6) Exploratory identification of laboratory change patterns based on key pre- and post-BP differences. These outcome settings were intended to align the study objectives with the level of evidence of a single-arm retrospective observational study, focusing on description and hypothesis generation rather than confirmatory efficacy inference.

### 2.6 Bias control

All potentially eligible consecutive cases were screened to reduce discretionary case selection. However, because valid paired laboratory measurements were required for the primary analyses, exclusion due to missing or unpaired post-BP data could still introduce selection bias. We therefore reported the number of paired observations for each biomarker and compared available baseline characteristics between the included cohort and patients excluded specifically because paired laboratory data were unavailable. In terms of evaluation strategy, the analysis was not limited to a single indicator but integrated laboratory indicators according to their biological properties into a multidimensional evaluation system covering lipid metabolism, inflammatory response, pancreatic injury, liver and kidney function, coagulation, and electrolyte balance. Furthermore, all analyses were strictly based on the original database using a standardized process, and sensitivity analyses were performed for skewed indicators to ensure the stability and robustness of the study conclusions.

### 2.7 Sample size

Because this study was a single-center retrospective observational study based on existing clinical data, the sample size was not prospectively estimated based on a preset effect size but was determined by the number of consecutive cases meeting the inclusion and exclusion criteria during the study period. To minimize selection bias, the principle of full-sample inclusion was adopted, and all evaluable cases during the study period were included, resulting in a final sample of 141 patients. Given that the primary purpose of this study was to describe the multisystem laboratory paired multisystem laboratory change patterns rather than to test a prespecified intervention effect, the sample size was primarily determined by the availability of case resources.

### 2.8 Statistical analysis

We used SPSS version 26.0 (IBM Corp., Armonk, NY, USA) for all analyses. Normality was checked with the Shapiro–Wilk test and Q-Q plots. Variables that followed an approximately normal distribution are shown as mean ± standard deviation and were compared using paired t-tests. Skewed variables, including triglycerides, IL-6, CRP, PCT, serum amylase, and ALT, are presented as median with interquartile range and were analysed with the Wilcoxon signed-rank test. Categorical data are reported as counts and percentages.

We calculated absolute paired changes (post minus pre) and relative percentage changes for each patient. For normally distributed differences, we used the t distribution to estimate 95% confidence intervals. For skewed differences, we used nonparametric bootstrap resampling with 1,000 iterations.

We performed exploratory stratified analyses based on BP modality and baseline biological burden (inflammation and lipid metabolism) where clinically meaningful. These were intended only to describe heterogeneity in laboratory changes, not to compare efficacy across modalities. Spearman rank correlation was used to assess relationships between changes in major laboratory indicators.

We constructed an exploratory, unvalidated multisystem laboratory change score to summarise directional pre-to-post BP changes across six prespecified domains: lipid metabolism, inflammation, pancreatic injury, renal function, liver function, and complete blood count/coagulation. Each domain contributed 1 point if its directional criterion was met (maximum score 6). The criteria were: a decrease in triglycerides and/or total cholesterol for lipid metabolism; a decrease in CRP, PCT, IL-6, and/or TNF-α for inflammation; a decrease in serum amylase for pancreatic injury; a decrease in creatinine and/or blood urea nitrogen for renal function; a decrease in total bilirubin and/or ALT for liver function; and a decrease in WBC, neutrophil percentage, and/or PT for complete blood count/coagulation. Albumin was excluded from the liver domain criterion because its short-term variation is heavily influenced by fluid administration, nutritional support, capillary leak, and replacement products. Electrolytes were analysed as a seventh domain but excluded from the six-domain score because potassium and calcium shifts are strongly affected by fluid resuscitation, electrolyte supplementation, and replacement solutions, making their directional interpretation less specific.

A domain was considered evaluable if at least one prespecified biomarker within it had a valid paired measurement. Missing biomarkers were not coded as favourable or unfavourable. If no valid paired biomarker was available for a domain, that domain was treated as non-evaluable rather than assigned a score of 0. Patients with non-evaluable domains were excluded from analyses requiring a complete 0 to 6 score.

Because a domain is credited with 1 point if any of its constituent biomarkers meets the directional criterion, domains containing multiple markers inherently have a higher probability of being scored than single-marker domains. For example, the inflammatory domain includes four markers (CRP, PCT, IL-6, TNF-α), whereas the pancreatic injury domain relies solely on serum amylase. This structural asymmetry is an inherent feature of the score and should be borne in mind when comparing domain-specific contributions or interpreting the total score. The score is intended solely for descriptive summarization, not for inferential comparisons across domains.

To test the robustness of this score, we examined two stricter sensitivity definitions. Under the first, a domain scored 1 only when at least 50% of its available component biomarkers changed in the prespecified direction. Under the second, a domain scored 1 only when all available component biomarkers changed in the prespecified direction. Single-marker domains retained the original criterion. These alternative definitions were used solely for sensitivity testing and were not intended as validated clinical thresholds.

We defined exploratory laboratory-change patterns using a prespecified rule-based approach rather than an unsupervised clustering algorithm. Twelve biomarkers were included: triglycerides, total cholesterol, IL-6, CRP, PCT, serum amylase, creatinine, blood urea nitrogen, ALT, total bilirubin, WBC, and PT. For each patient, we calculated the absolute relative percentage change for each available biomarker and used the median of these values as a summary measure. At least 8 of the 12 biomarkers were required to have valid paired measurements for pattern assignment; missing values were not imputed. Patients were then divided into three strata based on the cohort distribution of this summary measure: the upper tertile as the greater-change pattern, the middle tertile as intermediate, and the lower tertile as lower-change. Ties at the boundaries were kept within the same category, which could result in unequal group sizes. Because this procedure was descriptive and rule-based, no distance metric, dimensionality reduction, cluster optimisation, or stability assessment was applicable.

All analyses of individual laboratory variables used only available paired observations, so the effective sample size varied across biomarkers. We did not impute missing post-BP values. To assess potential selection bias, we compared baseline characteristics between included patients and those excluded due to missing or unpaired laboratory data.

We performed two additional sensitivity analyses. One restricted the sample to patients whose post-BP measurements were obtained within 24 h (*n* = 112) and descriptively compared the direction and magnitude of changes with the overall cohort; because sampling times remained heterogeneous even within this window, we treated this analysis as supportive rather than confirmatory. The other, as already noted, compared baseline characteristics between included and excluded patients to evaluate selection bias.

All tests were two-sided, with *P* < 0.05 considered statistically significant. Given the exploratory observational design, the large number of comparisons, and the absence of a control group, we interpreted *P*-values mainly as indicators of statistical signal strength and directional consistency, not as definitive confirmation. We did not adjust for multiple comparisons, so marginal *P*-values and weak correlations were interpreted cautiously, taking effect magnitude and clinical context into account.

## 3 Results

### 3.1 Baseline clinical characteristics and selection bias assessment

A total of 141 patients with AP were included in the primary analysis [97 males [68.79%], 44 females [31.21%]; mean age: 47.01 ± 14.53 years]. The cohort was enriched for hypertriglyceridemia-associated AP (HTG-AP, *n* = 96, 68.09%) and moderately severe to severe AP, representing the target population prioritized for BP at our institution. Biliary AP accounted for 27 cases (19.15%), alcohol-associated AP for 10 cases (7.09%), and other/idiopathic causes for 8 cases (5.67%). According to the revised Atlanta classification, 14 patients (9.93%) had mild AP, 55 (39.01%) had moderately severe AP, and 72 (51.06%) had severe AP. Organ failure was documented in 79 patients (56.03%), persistent organ failure in 61 (43.26%), and 65 patients (46.10%) required ICU admission. Hemoperfusion (HP) alone was administered to 81 patients (57.45%), single plasma exchange (PE) alone to 45 (31.91%), and combined BP modalities to 15 (10.64%). The median number of BP sessions was 2 (IQR 1∼3), and BP was initiated within 24 h of admission/diagnosis in 83 patients (58.87%). Baseline demographics, clinical features, and concomitant treatment details are summarized in Table 1.

To evaluate potential selection bias, baseline characteristics were compared between the included cohort (*n* = 141) and the 65 patients excluded specifically due to missing/unpaired post-BP laboratory data. No statistically significant differences were detected in age, sex, HTG-AP etiology, severe AP proportion, or ICU admission rates (all *P* > 0.05). However, excluded patients exhibited numerically higher rates of severe AP (61.54% vs. 51.06%) and ICU admission (58.46% vs. 46.10%), indicating that subtle selection bias toward clinically stable patients surviving to repeat testing cannot be completely ruled out. Therefore, clinically relevant selection bias toward patients who survived long enough and were sufficiently stable to undergo repeat laboratory testing cannot be excluded. Detailed comparisons are presented in Supplementary Table S1.

### 3.2 Sampling intervals between Pre- and post-BP measurements

The median interval between the pre-BP laboratory assessment and initiation of BP was 2.3 h (IQR 1.1∼4.8 h). The median interval from completion of BP to the first post-BP laboratory assessment was 11.6 h (IQR 6.2∼18.9 h). Post-BP measurements were obtained within 24 h in 112 patients (79.43%), between >24 and 48 h in 21 patients (14.89%), and after 48 h in 8 patients (5.67%). Although most measurements were obtained within the first 24 h, the actual timing remained heterogeneous because laboratory testing followed clinical needs rather than a standardized research schedule.

### 3.3 Overall magnitude and ranking of laboratory parameter changes

Within-patient paired comparisons demonstrated multidimensional shifts across lipid, inflammatory, pancreatic injury, hepatorenal, hematologic, and electrolyte domains.

#### 3.3.1 Ranking of change magnitudes

When ranked by median relative percentage change, the most pronounced reductions were concentrated in markers of inflammation, lipid metabolism, and pancreatic injury (Figures 2A–C). The largest relative decreases were observed for IL-6 (−86.79%), serum amylase (−86.67%), and triglycerides (−86.23%). Substantial reductions were also noted for TNF-α (−75.49%), ALT (−68.77%), CRP (−59.42%), PCT (−57.93%), and total cholesterol (−54.36%). Moderate shifts were observed in blood urea nitrogen (−44.87%), creatinine (−44.97%), WBC count (−44.54%), and total bilirubin (−42.63%), while neutrophil percentage (−16.75%), PT (−19.34%), and albumin (−11.11%) showed smaller relative changes. Ranking of change magnitudes and the multidimensional laboratory change pattern are shown in Figures 2A–C.

**Figure 2:**
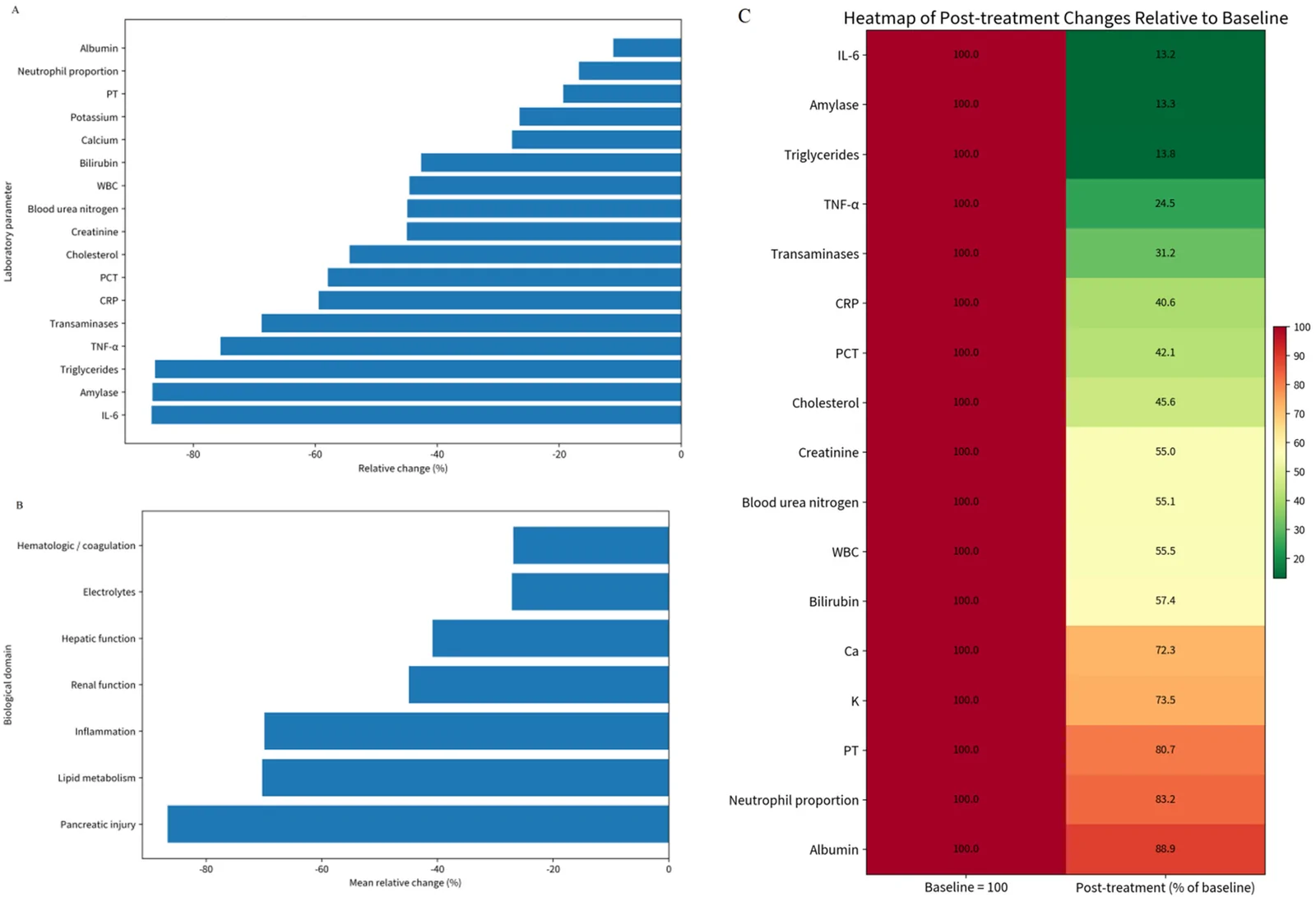
Multisystem laboratory changes before and after blood purification in patients With acute pancreatitis. **(A)** Ranking of relative changes in major laboratory parameters before and after blood purification. **(B)** Mean relative changes across different biological domains between pre- and post-BP measurements. **(C)** Heatmap of post-treatment changes in laboratory parameters relative to baseline.

#### 3.3.2 Sensitivity analyses

Non-parametric evaluation using Wilcoxon signed-rank tests and log-transformed paired t-tests confirmed that post-treatment reductions in highly skewed biomarkers (triglycerides, IL-6, PCT, and serum amylase) remained highly statistically significant (all *P* < 0.001; Table 2).

**Table 2:** Sensitivity analysis results.

Indicator	*P*-value from primary analysis (Wilcoxon signed-rank test)	*P*-value from sensitivity analysis (paired t-test)	*P*-value after log transformation
Triglycerides	<0.001	<0.001	<0.001
IL-6	<0.001	0.022	<0.001
PCT	<0.001	<0.001	<0.001
Serum amylase	<0.001	<0.001	<0.001

In the sub-analysis restricted to patients with post-BP sampling within 24 h (*n* = 112), relative percentage changes closely aligned with full-cohort findings: triglycerides decreased by a median of −84.90%, serum amylase by −84.70%, IL-6 by −83.80%, CRP by −57.10%, PCT by −55.40%, creatinine by −42.30%, and WBC count by −42.00% (Supplementary Table S2).

#### 3.3.3 Exploratory stratified analyses

In subgroup analyses stratified by BP modality and baseline biological burden, patients presenting with higher baseline parameter elevations demonstrated larger relative magnitude reductions (Table 3). For example, patients receiving combined therapy showed a greater median relative reduction in triglycerides (−90.01%) compared to HP alone (−65.91%) or PE alone (−72.25%) (*P* = 0.001). However, given the observational design and small combined-therapy subgroup (*n* = 15), these findings reflect baseline heterogeneity rather than comparative therapeutic superiority.

**Table 3:** Results of exploratory stratified analyses.

Stratification factor	Outcome indicator	Group with greater median relative change (median relative change, %)	Reference group (median relative change, %)	*P*-value
Blood purification modality	Triglycerides	Combined therapy group: −90.01	Hemoperfusion alone: −65.91; Single PE alone: −72.25	0.001
Blood purification modality	CRP	Hemoperfusion alone: −78.39	Single PE alone: −47.33; Combined therapy: −67.68	<0.001
Blood purification modality	Creatinine	Hemoperfusion alone: −30.28	Single PE alone: −27.97; Combined therapy: 1.32	0.009
Blood purification modality	Serum amylase	Hemoperfusion alone: −84.50	Single PE alone: −81.04; Combined therapy: −76.17	0.486
Baseline CRP stratification	CRP	High CRP group: −71.12	Low CRP group: −58.28	0.004
Baseline TG stratification	Triglycerides	High TG group: −87.55	Low TG group: −45.45	<0.001
Baseline TG stratification	Cholesterol	High TG group: −62.41	Low TG group: −21.28	<0.001
Baseline creatinine strat.	Creatinine	High creatinine group: −49.94	Low creatinine group: −10.12	<0.001
Baseline creatinine strat.	Blood urea nitrogen	High creatinine group: −45.16	Low creatinine group: −34.55	0.022

Due to the small sample size of the combined therapy group (*n* = 15), the median relative changes in this subgroup are provided for reference only and should not be used for confirmatory statistical inference.

#### 3.3.4 Correlation analysis across biomarker changes

Correlation matrix analysis revealed strong intra-domain consistency, such as between paired creatinine and BUN changes (Spearman r = 0.726, *P* < 0.001; Figure 3). Weak positive correlations were noted between serum amylase and PCT changes (r = 0.205, *P* = 0.028), while an inverse correlation was observed between triglyceride and serum amylase changes (r = −0.174, *P* = 0.045). In contrast, cross-domain shifts between triglycerides and CRP (r = 0.145, *P* = 0.096) or CRP and creatinine (r = 0.026, *P* = 0.756) were not statistically correlated, indicating asynchronous multi-system kinetics.

**Figure 3:**
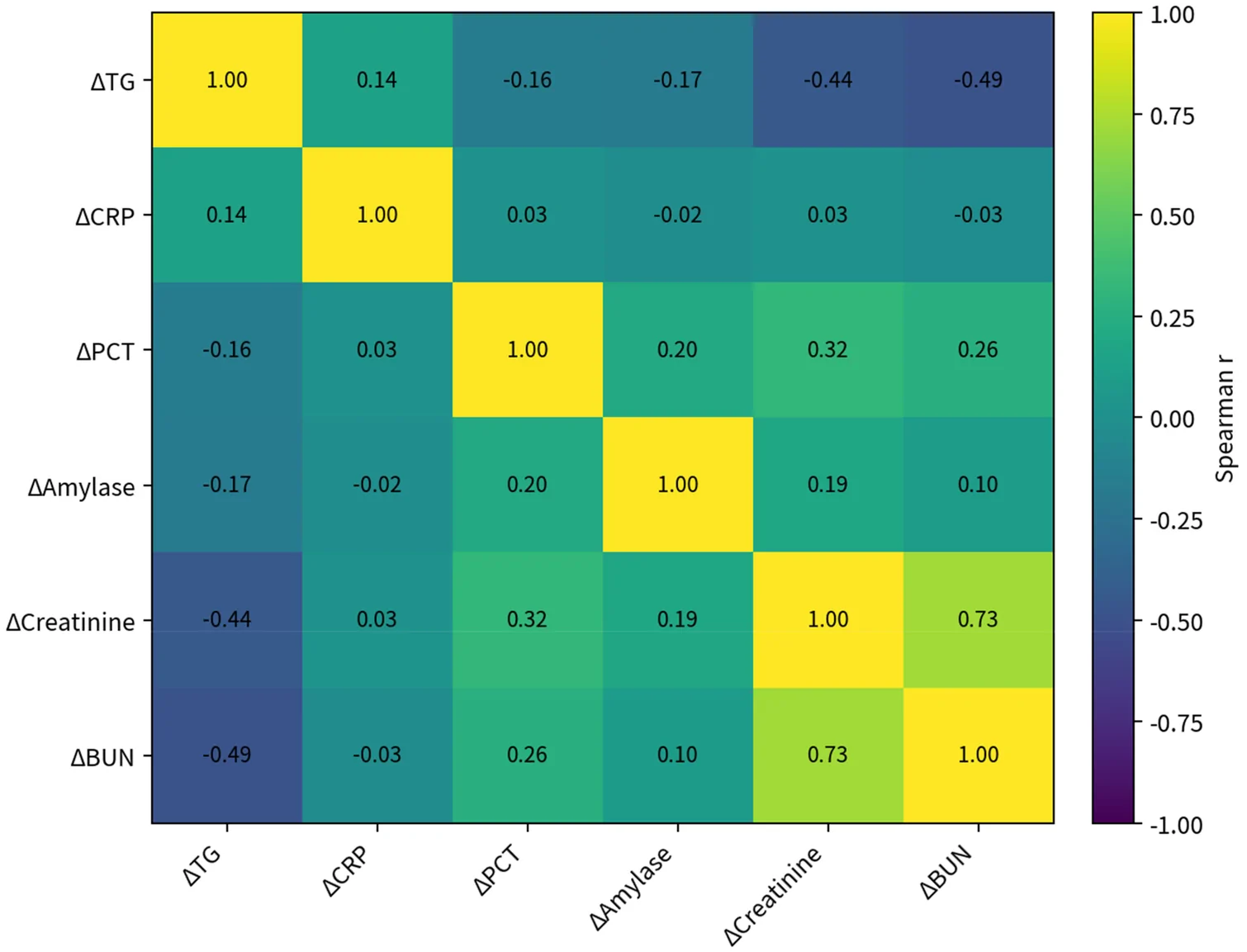
Correlation matrix of changes in major laboratory parameters.

#### 3.3.5 Descriptive multisystem laboratory change score

The primary exploratory multisystem laboratory change score was 4.24 ± 1.34, with a median of 4 (IQR 4∼5) and a range of 0∼6. Directional-change criteria were met in the pancreatic injury domain in 121 patients (85.82%), lipid metabolism in 118 (83.69%), complete blood count/coagulation in 111 (78.72%), inflammation in 109 (77.30%), renal function in 102 (72.34%), and liver function in 89 (63.12%). Overall, 124 patients (87.94%) met the directional-change criterion in at least 3 domains, 100 (70.92%) in at least 4 domains, and 29 (20.57%) in all 6 domains (Table 4). High directional consistency was observed in pancreatic injury (85.82%), lipid metabolism (83.69%), and hematology/coagulation (78.72%) domains, followed by inflammation (77.30%), renal function (72.34%), and hepatic function (63.12%). Overall, 124 patients (87.94%) displayed concurrent directional changes across ≥3 domains, 100 (70.92%) across ≥4 domains, and 29 (20.57%) across all 6 scoring domains. Mean scores across treatment modalities were 4.08 ± 1.31 for HP alone, 4.31 ± 1.27 for PE alone, and 4.73 ± 1.39 for combined therapy.

**Table 4:** Prespecified algorithm for the exploratory, unvalidated multisystem laboratory change score.

Dimension	Representative indicators	Scoring rule	Meeting directional-change criterion *n* (%)
Lip metabolism	Triglycerides, total cholesterol	1 point if at least one indicator shows prespecified directional change from the pre-BP to the post-BP assessment	118 (83.69)
Inflammatory response	CRP, PCT, IL-6, TNF-α	1 point if at least one indicator shows prespecified directional change from the pre-BP to the post-BP assessment	109 (77.30)
Pancreatic injury	Serum amylase	1 point if serum amylase decreased from the pre-BP to the post-BP assessment	121 (85.82)
Renal function	Blood urea nitrogen, creatinine	1 point if at least one indicator shows prespecified directional change from the pre-BP to the post-BP assessment	102 (72.34)
Liver function	Total bilirubin, ALT	1 point if at least one indicator shows prespecified directional change from the pre-BP to the post-BP assessment	89 (63.12)
CBC/coagulation	WBC, neutrophil percentage, PT	1 point if at least one indicator shows prespecified directional change from the pre-BP to the post-BP assessment	111 (78.72)

Under the stricter ≥50%-of-available-markers definition, the mean score decreased to approximately 3.71 ± 1.42; 110 patients (78.01%) met criteria in ≥3 domains, 82 (58.16%) in ≥4 domains, and 48 (34.04%) in ≥5 domains. Under the strictest all-available-markers definition, the mean score further decreased to approximately 3.18 ± 1.39; 91 patients (64.54%) met criteria in ≥3 domains, 61 (43.26%) in ≥4 domains, and 30 (21.28%) in ≥5 domains. The reduction in score prevalence under stricter definitions confirms that the primary score is sensitive to the domain-level rule and should therefore be regarded as an exploratory, unvalidated descriptive summary.

#### 3.3.6 Exploratory stratification of laboratory change patterns

Rule-based stratification based on the patient-level median absolute relative change across the 12 prespecified biomarkers identified three exploratory laboratory-change patterns: greater-change (*n* = 40), intermediate-change (*n* = 67), and lower-change (*n* = 34). The groups corresponded to the upper, middle, and lower thirds of the cohort-level distribution, respectively; unequal group sizes resulted from tied values at the tertile boundaries (Figure 4). Patients in the greater-change group showed larger relative changes across lipid, inflammatory, pancreatic injury, and selected organ-function parameters, whereas patients in the lower-change group showed smaller changes across most dimensions. These categories were generated solely for descriptive visualization of within-cohort heterogeneity and should not be interpreted as biological phenotypes, treatment-response clusters, or evidence of differential BP efficacy.

**Figure 4:**
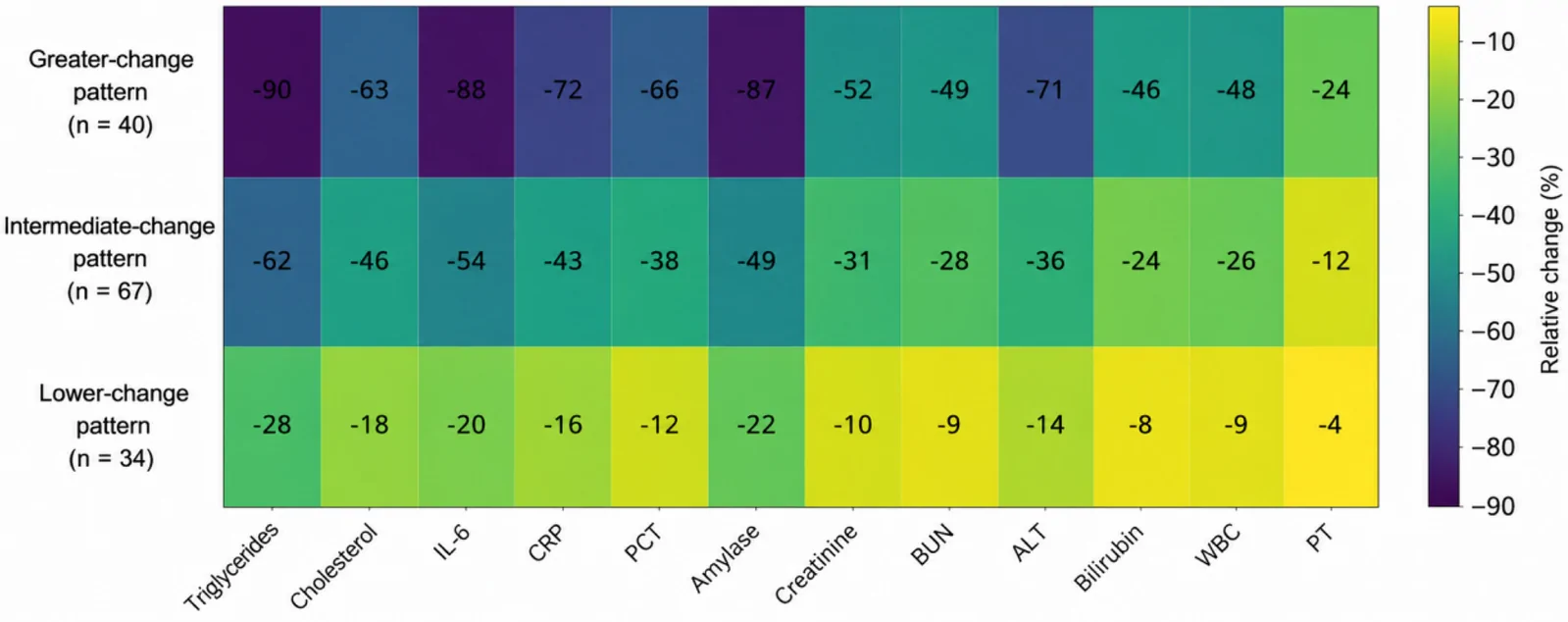
Exploratory rule-based patterns of relative laboratory changes observed before and after blood purification in patients with acute pancreatitis.

Patients were categorized according to the cohort distribution of their median absolute relative change across 12 prespecified laboratory biomarkers. The greater-change, intermediate-change, and lower-change groups comprised 40, 67, and 34 patients, respectively. The heatmap displays signed relative changes for descriptive visualization, whereas pattern assignment was based on the absolute magnitude of patient-level relative changes. These groups represent exploratory descriptive patterns and should not be interpreted as biological phenotypes or therapeutic response categories.

### 3.4 Detailed biological shifts by domain

#### 3.4.1 Lipid, inflammatory, and pancreatic injury biomarkers

Median and IQR values for baseline and post-BP measurements across lipid, inflammatory, and pancreatic injury parameters are detailed in Table 5. Significant post-treatment decreases were observed for total cholesterol, triglycerides, CRP, PCT, IL-6, and serum amylase (all *P* < 0.05). TNF-α exhibited a decreasing trend that did not reach statistical significance (*P* = 0.111).

**Table 5:** Comparison of lipid, inflammatory, and pancreatic injury-related indicators before and after blood purification.

Indicator	Paired n	Pre-treatment (median, IQR)	Post-treatment (median, IQR)	Median Abs. Change (*Δ*)	Median Rel. Change (%)	95% CI for median relative change	P-value
Cholesterol (mmol/L)	136	7.82 (4.50, 11.90)	3.95 (2.90, 5.10)	−3.87	−54.36		<0.001
Triglycerides (mmol/L)	139	16.50 (8.20, 32.80)	2.27 (1.40, 4.10)	−14.23	−86.23	(-91.35, −81.11)	<0.001
CRP (mg/L)	132	142.50 (78.20, 215.00)	51.20 (22.80, 92.40)	−91.3	−59.42	(−74.30, −44.54)	<0.001
PCT (ng/mL)	121	2.85 (0.82, 9.40)	1.20 (0.35, 3.80)	−1.65	−57.93	(−58.70, −57.16)	<0.001
IL-6 (pg/mL)	94	215.40 (68.50, 680.20)	28.40 (12.10, 85.30)	−187	−86.79	(−241.79, 68.21)	<0.001
TNF-α (pg/mL)	82	3.12 (1.20, 8.50)	1.85 (0.90, 3.20)	−1.27	−75.49		0.111
Serum amylase (U/L)	137	780.00 (310.00, 1650.00)	104.00 (52.00, 210.00)	−676	−86.67	(−119.83, −53.51)	<0.001

Values are presented as Median (IQR) due to skewed distributions. Absolute change (*Δ*) and Relative change (%) represent median within-patient paired differences. Paired *n* represents the number of patients with valid paired pre- and post-BP measurements. CRP: C-reactive protein; PCT: procalcitonin; IL-6: interleukin-6; TNF-α: tumor necrosis factor-alpha.

#### 3.4.2 Hepatorenal, hematologic, coagulation, and electrolyte parameters

Hepatopancreatic, renal, complete blood count, coagulation, and electrolyte changes are detailed in Table 6. WBC count decreased from 17.26 ± 7.38 to 9.57 ± 6.42 × 10^9^/L (*P* < 0.001), neutrophil percentage from 87.70 ± 8.37% to 73.01 ± 11.03% (*P* < 0.001), and PT from 17.82 ± 9.63 to 14.38 ± 9.88 s (*P* = 0.002). Serum creatinine and BUN dropped by medians of −44.97% and −44.87%, respectively (*P* < 0.001). Electrolyte shifts (potassium and calcium) were observed but remain heavily confounded by exogenous fluid and replacement protocols.

**Table 6:** Comparison of liver and kidney function, complete blood count, coagulation, and electrolyte indicators before and after blood purification.

Indicator	Paired n	Pre-treatment	Post-treatment	Absolute Change (*Δ*)	Relative Change (%)	95% CI for median relative change (bootstrap/ Mean paired change (95% CI)	P-value
Blood urea nitrogen (mmol/L)^†^	139	8.20 (5.10, 14.30)	4.80 (3.20, 7.60)	−3.4	−44.87	(−45.79, −43.96)^†^	<0.001
Creatinine (*μ*mol/L)^†^	140	148.00 (82.00, 310.00)	81.50 (58.00, 152.00)	−66.5	−44.97	(−64.49, −25.45)^†^	<0.001
Total Bilirubin (μmol/L)^†^	134	28.40 (16.20, 58.90)	16.30 (10.50, 32.10)	−12.1	−42.63	(−47.22, −38.04)^†^	<0.001
ALT (U/L)^†^	133	88.00 (35.00, 260.00)	27.50 (16.00, 64.00)	−60.5	−68.77	(−79.00, −58.54)^†^	<0.001
Albumin (g/L)^‡^	129	40.20 ± 21.48	35.73 ± 5.86	−4.47	−11.11	*−4.47 g/L (−7.64 to −1.30)* ^‡^	0.011
WBC (×10⁹/L)^‡^	140	17.26 ± 7.38	9.57 ± 6.42	−7.69	−44.54	*−7.69* *×* *10⁹/L (−8.55 to −6.83)* ^‡^	<0.001
Neutrophil percentage (%)^‡^	138	87.70 ± 8.37	73.01 ± 11.03	−14.69	−16.75	*−14.69 percentage points (−16.07 to −13.31)* ^‡^	<0.001
PT (s)^‡^	126	17.82 ± 9.63	14.38 ± 9.88	−3.45	−19.34	*−3.45 s (−4.17 to −2.73)* ^‡^	0.002
Potassium (mmol/L)^‡^	139	4.24 ± 0.96	3.12 ± 1.24	−1.12	−26.49	*−1.12 mmol/L (−1.26 to −0.98)* ^‡^	<0.001
Calcium (mmol/L)^‡^	136	3.01 ± 1.16	2.18 ± 0.30	−0.83	−27.72	*−0.83 mmol/L (−0.95 to −0.71)* ^‡^	<0.001

Values are presented as Median (IQR) for skewed variables or Mean ± SD for normally distributed variables.

† Skewed variables: median absolute/relative changes are shown; 95% confidence intervals for the median relative change were estimated via nonparametric bootstrap resampling (1,000 iterations, percentile method).

‡ Normally distributed variables: mean paired change and its 95% confidence interval are presented.

## 4 Discussion

In this retrospective single-arm observational study of 141 consecutive patients with AP who underwent BP, we integrated multidimensional laboratory data to map paired biomarker trajectories across seven biological domains. The primary analysis revealed marked numerical reductions in several key parameters most notably triglycerides, IL-6, serum amylase, CRP, PCT, and ALT. However, because all patients simultaneously received standardized supportive management and no contemporaneous non-BP control group was present, these shifts must be interpreted strictly as temporal laboratory changes during the clinical course, rather than evidence of BP efficacy or causal treatment effects (13, 14). Modern clinical paradigms increasingly recognize AP not as a localized pancreatic insult, but as a dynamic, multisystem inflammatory and metabolic syndrome; evaluating multidimensional laboratory trajectories therefore aligns closely with real-world disease evolution (13, 14).

Indicators of lipid toxicity burden (triglycerides, total cholesterol) and pancreatic enzyme activity (serum amylase) exhibited some of the largest relative percentage reductions in this cohort. Elevated baseline triglycerides are well-established drivers of persistent organ failure, necrotizing pancreatitis, and systemic complications (15–17). The pronounced drop in triglycerides observed here aligns with clinical expectations for BP utilization in hypertriglyceridemia-associated AP (HTG-AP) (15–17). Nevertheless, these changes reflect observed laboratory fluctuations under multi-modal care and must not be directly equated with clinical outcome improvement (15–17).

A crucial context for interpreting these results is the population profile of our institution. Hypertriglyceridemia represented the primary etiology in 68.09% of cases, with a mean baseline triglyceride level of 25.80 ± 32.21 mmol/L. At our center, severe hypertriglyceridemia is a principal indication for initiating hemoperfusion or plasma exchange. Consequently, the high magnitude of triglyceride clearance and associated multi-domain patterns reported here are representative of a heavily enriched, lipid-driven AP cohort and cannot be directly generalized to unselected AP populations with predominantly biliary or alcoholic etiologies (15–17).

Inflammatory mediators (IL-6, CRP, PCT) showed prominent temporal decreases, whereas TNF-α displayed a non-significant decreasing trend. Both IL-6 and CRP serve as established biomarkers for severity stratification and risk prediction in AP, with IL-6 offering high predictive accuracy for infected pancreatic necrosis and organ failure (18, 19). However, circulating inflammatory burden in AP is heavily influenced by disease progression, fluid resuscitation, anti-infective therapy, and organ support; thus, observed declines cannot be isolated as specific anti-inflammatory effects of BP (18, 19).

Similarly, renal indices (BUN, creatinine) demonstrated moderate dynamic shifts, underscoring their sensitivity to systemic hemodynamic restoration and fluid resuscitation (20–22). Conversely, serum albumin changes were subtle; given that albumin concentrations are confounded by capillary leak, exogenous volume administration, and replacement fluid compositions, albumin fluctuations represent a composite reflection of internal environmental shifts rather than a clean signal of clinical benefit or deterioration (22, 23). Hematologic and coagulation parameters (WBC, neutrophil percentage, PT) displayed synchronized moderate declines, supporting the paradigm that AP involves a closely linked inflammatory-coagulation network (24, 25). However, coagulation parameters showed smaller relative magnitudes of change than lipid or inflammatory markers, suggesting smaller short-term relative changes than lipid or inflammatory markers (24, 25).

Beyond single-indicator evaluations, we introduced an exploratory multisystem change score, directional-change consistency analysis, and rule-based pattern stratification. Over 87% of patients demonstrated concurrent directional shifts across at least three biological domains, reinforcing the notion that post-treatment laboratory shifts reflect a composite, cross-system pattern rather than isolated biomarker noise. Correlation matrix analysis demonstrated strong intra-domain coupling (e.g., BUN and creatinine, *r* = 0.726), but weak or non-significant cross-domain correlations (e.g., triglycerides vs. CRP, or CRP vs. creatinine). This indicates that while multidimensional shifts occur concurrently, individual biological domains evolve with asynchronous, non-linear kineticsa finding consistent with recent real-world evaluations of therapeutic plasma exchange and critical care blood purification (26–28).

Focusing on multidimensional laboratory trajectories provides a realistic observational endpoint for single-arm retrospective studies where hard clinical endpoints (e.g., mortality, ICU length of stay) are heavily confounded (14, 28, 29). Emerging AP risk scores (e.g., BUN/Alb, CRP/Alb, TBil/Alb) similarly emphasize integrated composite markers over single static tests (14, 28, 29). The analytical strategy deployed here offers a reproducible framework for mapping real-world laboratory heterogeneity, generating testable hypotheses for future prospectively controlled trials.

The findings of this study must be interpreted within the context of several major methodological limitations: first, the single-center, retrospective, single-arm design without a parallel control group constitutes the primary limitation. Observed pre- to post-treatment differences cannot be causally attributed to BP, as they reflect the combined influence of natural disease resolution, intensive fluid resuscitation, metabolic care, and organ support. Second, non-standardized laboratory sampling schedules introduce temporal kinetics bias. Biomarkers exhibit vastly different clearance rates: triglycerides and amylase alter rapidly, whereas CRP, PCT, and renal parameters evolve over longer, highly variable timeframes. Although 79.43% of post-BP blood samples were drawn within 24 h, subtle differences in sampling intervals (e.g., 4 h vs. 18 h post-BP) complicate direct kinetic comparisons across parameters and domains. Third, selection bias and missing data mechanisms require cautious consideration. Out of 246 screened patients, 65 were excluded due to missing paired post-treatment data. Although baseline demographics between included and excluded patients showed no statistically significant differences, excluded cases had numerically higher severe AP (61.54% vs. 51.06%) and ICU admission rates (58.46% vs. 46.10%). This introduces potential selection bias toward patients who survived long enough and were sufficiently stable to complete repeat testing. Fourth, substantial subgroup imbalances existed across BP modalities (HP alone: *n* = 81; PE alone: *n* = 45; combined: *n* = 15), driven by clinical indication rather than random assignment. Subgroup stratification results are therefore exploratory and must not be construed as comparative effectiveness evidence. Furthermore, the exploratory multisystem change score is structurally weighted toward multidomain markers because its ‘any-marker’ rule gives a higher chance of scoring to domains with more component biomarkers. This unvalidated score should therefore be interpreted only as a crude descriptive aggregate, and its domain-wise contributions should not be directly compared. Finally, the multisystem change score and laboratory change patterns are purely descriptive, unvalidated analytical tools. They were developed to summarize complex real-world data rather than to establish clinical efficacy or prognostic utility.

In conclusion, in this clinically enriched cohort of 141 BP-treated AP patients with high baseline hypertriglyceridemia, paired pre- and post-treatment laboratory assessments demonstrated multidimensional reductions across lipid, inflammatory, pancreatic injury, and hepatorenal domains. These descriptive shifts reflect composite real-world clinical trajectories under multi-modal care. Multicenter prospective controlled studies with protocolized, standardized sampling timelines are required to establish the causal contribution of blood purification relative to standard supportive care.

## Data Availability

The raw data supporting the conclusions of this article will be made available by the authors, without undue reservation. The analytical code used for all statistical analyses, sensitivity analyses, pattern stratification, and figure generation is provided as Supplementary File S1.
