# Supplementary material for: Multisystem laboratory changes before and after blood purification in acute pancreatitis: a real-world descriptive analysis of 141 cases
Source: Front Med (Lausanne). 2026 Sep 23;13:1850265. doi: 10.3389/fmed.2026.1850265 (PMC13642849; doi:10.3389/fmed.2026.1850265)
Supplement: Supplementary file 1 [file Datasheet1.pdf]

Supplementary File S1

Analytical Code

Multisystem Laboratory Changes Before and After Blood Purification in Acute Pancreatitis: A Real-World Descriptive Analysis of 141 Cases

This supplementary file provides reproducible R code corresponding to the statistical procedures described in the manuscript, including paired analyses, bootstrap confidence intervals, subgroup analyses, correlation analysis, the exploratory multisystem laboratory change score, stricter score sensitivity definitions, rule-based laboratory-change pattern stratification, selection-bias comparisons, time-window sensitivity analyses, and figure generation.

Software: R version 4.3.1 or later. Package versions should be recorded at the time of final analysis using sessionInfo().

Important: variable names below are standardized analysis names. They should be mapped to the corresponding variables in the de-identified study dataset before execution. No patient identifiers are required.

1. Packages and reproducibility setup

```
# ============================================================
# Supplementary File S1 - Analytical code
# R version 4.3.1 or later
# ============================================================

set.seed(20260827)

required_pkgs <- c(
  "tidyverse", "readxl", "boot", "broom", "rstatix",
  "ggplot2", "pheatmap", "Hmisc"
)

to_install <- required_pkgs[!required_pkgs %in% rownames(installed.packages())]
if (length(to_install) > 0) install.packages(to_install)

invisible(lapply(required_pkgs, library, character.only = TRUE))

# Record software/package versions for reproducibility
sessionInfo()
```

2. Import data and define analysis variables

```
# Expected structure: one row per patient.
# Replace the file name and variable names with the final de-identified dataset.

dat <- readxl::read_excel("AP_BP_analysis_dataset.xlsx")

# Core identifiers and clinical variables expected:
# id
# included                    1 = included in primary cohort, 0 = excluded
# excluded_missing_pair       1 = excluded because paired laboratory data unavailable
# age
# sex                         e.g., "Male"/"Female"
# htg_ap                      1/0
# severe_ap                   1/0
# icu                         1/0
# bp_modality                 "HP", "PE", "Combined"
# bp_start_hours              hours from admission/diagnosis to BP initiation
# post_bp_hours               hours from BP completion to principal post-BP blood draw
#
# Laboratory variables use suffixes "_pre" and "_post":
# cholesterol, tg, crp, pct, il6, tnfa, amylase, bun, creatinine,
# bilirubin, alt, albumin, wbc, neutrophil_pct, pt, potassium, calcium

primary <- dat %>% filter(included == 1)

lab_vars <- c(
```

```
  "cholesterol","tg","crp","pct","il6","tnfa","amylase",
  "bun","creatinine","bilirubin","alt","albumin","wbc",
  "neutrophil_pct","pt","potassium","calcium"
)

# Helper functions
pct_change <- function(pre, post) {
  ifelse(is.na(pre) | is.na(post) | pre == 0, NA_real_,
         (post - pre) / pre * 100)
}

abs_change <- function(pre, post) {
  ifelse(is.na(pre) | is.na(post), NA_real_, post - pre)
}

for (v in lab_vars) {
  primary[[paste0(v, "_delta")]] <-
    abs_change(primary[[paste0(v, "_pre")]], primary[[paste0(v, "_post")]])
  primary[[paste0(v, "_pct")]] <-
    pct_change(primary[[paste0(v, "_pre")]], primary[[paste0(v, "_post")]])
}
```

3. Sampling-interval summaries

```
# Pre-BP and post-BP sampling interval summaries
# If a separate variable for pre-BP draw-to-BP initiation exists, use it here.

primary %>%
  summarise(
    n = n(),
    post_bp_median_h = median(post_bp_hours, na.rm = TRUE),
    post_bp_q1_h = quantile(post_bp_hours, 0.25, na.rm = TRUE),
    post_bp_q3_h = quantile(post_bp_hours, 0.75, na.rm = TRUE),
    within_24h = sum(post_bp_hours <= 24, na.rm = TRUE),
    gt24_to_48h = sum(post_bp_hours > 24 & post_bp_hours <= 48, na.rm = TRUE),
    gt48h = sum(post_bp_hours > 48, na.rm = TRUE)
  )

primary <- primary %>%
  mutate(
    post_sampling_group = case_when(
      post_bp_hours <= 24 ~ "<=24 h",
      post_bp_hours > 24 & post_bp_hours <= 48 ~ ">24-48 h",
      post_bp_hours > 48 ~ ">48 h",
      TRUE ~ NA_character_
    )
  )

table(primary$post_sampling_group, useNA = "ifany")
prop.table(table(primary$post_sampling_group))
```

4. Paired descriptive analyses and matched sample sizes

```
# For each biomarker:
# - matched n
# - distribution of paired differences
# - paired t-test if approximately normal
# - Wilcoxon signed-rank test if markedly skewed
# - patient-level median relative change
# - 95% CI for paired mean/median change

bootstrap_median_ci <- function(x, R = 1000, conf = 0.95) {
  x <- x[is.finite(x)]
  if (length(x) < 5) return(c(NA_real_, NA_real_))
  b <- boot::boot(x, statistic = function(d, i) median(d[i], na.rm = TRUE), R = R)
  ci <- tryCatch(boot::boot.ci(b, conf = conf, type = "perc"),
                 error = function(e) NULL)
  if (is.null(ci) || is.null(ci$percent)) return(c(NA_real_, NA_real_))
  ci$percent[4:5]
}

paired_results <- purrr::map_dfr(lab_vars, function(v) {
```

```r
  pre  <- primary[[paste0(v, "_pre")]]
  post <- primary[[paste0(v, "_post")]]
  cc   <- complete.cases(pre, post)
  pre  <- pre[cc]
  post <- post[cc]
  d    <- post - pre
  rpct <- ifelse(pre == 0, NA_real_, d / pre * 100)

  shapiro_p <- if (length(d) >= 3 && length(d) <= 5000)
    shapiro.test(d)$p.value else NA_real_

  # Normality rule can be refined using Q-Q plots in addition to Shapiro-Wilk.
  normal_diff <- !is.na(shapiro_p) && shapiro_p >= 0.05

  if (normal_diff) {
    test <- t.test(post, pre, paired = TRUE)
    ci_l <- test$conf.int[1]
    ci_u <- test$conf.int[2]
    pval <- test$p.value
    effect <- mean(d, na.rm = TRUE)
    summary_type <- "Mean paired change"
  } else {
    test <- wilcox.test(post, pre, paired = TRUE, exact = FALSE)
    ci <- bootstrap_median_ci(d, R = 1000)
    ci_l <- ci[1]
    ci_u <- ci[2]
    pval <- test$p.value
    effect <- median(d, na.rm = TRUE)
    summary_type <- "Median paired change"
  }

  tibble(
    indicator = v,
    paired_n = length(d),
    shapiro_p_difference = shapiro_p,
    analysis = ifelse(normal_diff, "Paired t-test", "Wilcoxon signed-rank test"),
    effect_label = summary_type,
    paired_change = effect,
    ci95_lower = ci_l,
    ci95_upper = ci_u,
    median_relative_change_pct = median(rpct, na.rm = TRUE),
    p_value = pval
  )
})

paired_results
```

5. Distribution summaries for manuscript Tables 5 and 6

```r
# Skewed biomarkers reported as Median (IQR)
skewed_vars <- c(
  "cholesterol","tg","crp","pct","il6","tnfa","amylase",
  "bun","creatinine","bilirubin","alt"
)

# Approximately normal biomarkers reported as Mean +/- SD
normal_vars <- c("albumin","wbc","neutrophil_pct","pt","potassium","calcium")

summarise_lab <- function(v) {
  pre <- primary[[paste0(v, "_pre")]]
  post <- primary[[paste0(v, "_post")]]
  cc <- complete.cases(pre, post)
  pre <- pre[cc]; post <- post[cc]

  if (v %in% skewed_vars) {
    tibble(
      indicator = v,
      paired_n = length(pre),
      pre_summary = sprintf("%.2f (%.2f, %.2f)",
                            median(pre), quantile(pre,.25), quantile(pre,.75)),
      post_summary = sprintf("%.2f (%.2f, %.2f)",
                             median(post), quantile(post,.25), quantile(post,.75))
```

```r
    )
  } else {
    tibble(
      indicator = v,
      paired_n = length(pre),
      pre_summary = sprintf("%.2f +/- %.2f", mean(pre), sd(pre)),
      post_summary = sprintf("%.2f +/- %.2f", mean(post), sd(post))
    )
  }
}

table_summary <- purrr::map_dfr(lab_vars, summarise_lab) %>%
  left_join(paired_results, by = "indicator")

table_summary
```

6. Exploratory subgroup analyses

```r
# BP modality subgroup analysis
# Kruskal-Wallis is used here for relative-change distributions because
# these measures are typically skewed.

subgroup_vars <- c("tg_pct", "crp_pct", "creatinine_pct", "amylase_pct")

bp_subgroup_results <- purrr::map_dfr(subgroup_vars, function(v) {
  x <- primary %>%
    select(bp_modality, all_of(v)) %>%
    filter(!is.na(bp_modality), !is.na(.data[[v]]))

  kw <- kruskal.test(reformulate("bp_modality", response = v), data = x)

  x %>%
    group_by(bp_modality) %>%
    summarise(
      n = n(),
      median_relative_change = median(.data[[v]], na.rm = TRUE),
      .groups = "drop"
    ) %>%
    mutate(indicator = v, overall_p = kw$p.value)
})

bp_subgroup_results

# Example baseline burden strata using cohort medians.
# If manuscript-defined clinical cutoffs were used, replace these median cutoffs.

primary <- primary %>%
  mutate(
    crp_burden = if_else(crp_pre >= median(crp_pre, na.rm = TRUE), "High", "Low"),
    tg_burden = if_else(tg_pre >= median(tg_pre, na.rm = TRUE), "High", "Low"),
    creatinine_burden = if_else(
      creatinine_pre >= median(creatinine_pre, na.rm = TRUE), "High", "Low"
    )
  )

wilcox.test(tg_pct ~ tg_burden, data = primary, exact = FALSE)
wilcox.test(cholesterol_pct ~ tg_burden, data = primary, exact = FALSE)
wilcox.test(crp_pct ~ crp_burden, data = primary, exact = FALSE)
wilcox.test(creatinine_pct ~ creatinine_burden, data = primary, exact = FALSE)
wilcox.test(bun_pct ~ creatinine_burden, data = primary, exact = FALSE)
```

7. Correlation analysis of biomarker changes

```r
corr_vars <- c(
  "tg_delta", "crp_delta", "pct_delta",
  "amylase_delta", "creatinine_delta", "bun_delta"
)

corr_dat <- primary %>% select(all_of(corr_vars))

# Spearman correlation coefficients
corr_r <- Hmisc::rcorr(as.matrix(corr_dat), type = "spearman")$r
```

```
corr_p <- Hmisc::rcorr(as.matrix(corr_dat), type = "spearman")$P

corr_r
corr_p

# Selected correlations reported in the manuscript
cor.test(primary$creatinine_delta, primary$bun_delta,
         method = "spearman", exact = FALSE, use = "complete.obs")
cor.test(primary$amylase_delta, primary$pct_delta,
         method = "spearman", exact = FALSE, use = "complete.obs")
cor.test(primary$tg_delta, primary$amylase_delta,
         method = "spearman", exact = FALSE, use = "complete.obs")
cor.test(primary$tg_delta, primary$crp_delta,
         method = "spearman", exact = FALSE, use = "complete.obs")
cor.test(primary$crp_delta, primary$creatinine_delta,
         method = "spearman", exact = FALSE, use = "complete.obs")
```

8. Exploratory multisystem laboratory change score

```
# Directional criterion: decrease from pre-BP to post-BP.
# A domain is evaluable if at least one prespecified biomarker has a valid pair.
# Missing biomarkers are not coded as favorable or unfavorable.
# If a domain has no valid paired biomarker, it is non-evaluable.

is_decrease <- function(pre, post) {
  ifelse(is.na(pre) | is.na(post), NA, post < pre)
}

primary <- primary %>%
  mutate(
    chol_dec = is_decrease(cholesterol_pre, cholesterol_post),
    tg_dec   = is_decrease(tg_pre, tg_post),
    crp_dec  = is_decrease(crp_pre, crp_post),
    pct_dec  = is_decrease(pct_pre, pct_post),
    il6_dec  = is_decrease(il6_pre, il6_post),
    tnfa_dec = is_decrease(tnfa_pre, tnfa_post),
    amy_dec  = is_decrease(amylase_pre, amylase_post),
    bun_dec  = is_decrease(bun_pre, bun_post),
    cr_dec   = is_decrease(creatinine_pre, creatinine_post),
    bili_dec = is_decrease(bilirubin_pre, bilirubin_post),
    alt_dec  = is_decrease(alt_pre, alt_post),
    wbc_dec  = is_decrease(wbc_pre, wbc_post),
    neut_dec = is_decrease(neutrophil_pct_pre, neutrophil_pct_post),
    pt_dec   = is_decrease(pt_pre, pt_post)
  )

domain_score_any <- function(...) {
  x <- c(...)
  if (all(is.na(x))) return(NA_real_)
  as.numeric(any(x %in% TRUE, na.rm = TRUE))
}

primary$score_lipid <- purrr::pmap_dbl(
  primary[c("tg_dec","chol_dec")], domain_score_any
)
primary$score_inflammation <- purrr::pmap_dbl(
  primary[c("crp_dec","pct_dec","il6_dec","tnfa_dec")], domain_score_any
)
primary$score_pancreas <- purrr::pmap_dbl(
  primary[c("amy_dec")], domain_score_any
)
primary$score_renal <- purrr::pmap_dbl(
  primary[c("cr_dec","bun_dec")], domain_score_any
)
primary$score_liver <- purrr::pmap_dbl(
  primary[c("bili_dec","alt_dec")], domain_score_any
)
primary$score_cbc_coag <- purrr::pmap_dbl(
  primary[c("wbc_dec","neut_dec","pt_dec")], domain_score_any
)

score_cols <- c(
```

```
  "score_lipid","score_inflammation","score_pancreas",
  "score_renal","score_liver","score_cbc_coag"
)

primary$score_complete <- rowSums(!is.na(primary[score_cols])) == 6
primary$score_primary <- ifelse(
  primary$score_complete,
  rowSums(primary[score_cols], na.rm = FALSE),
  NA_real_
)

summary(primary$score_primary)
table(primary$score_primary, useNA = "ifany")

# Domain-level directional-change prevalence
primary %>%
  summarise(across(all_of(score_cols),
                   ~ sum(.x == 1, na.rm = TRUE))) %>%
  pivot_longer(everything(), names_to = "domain", values_to = "n_meeting")
```

9. Stricter score sensitivity definitions

```
domain_score_prop <- function(x, threshold = 0.5, require_all = FALSE) {
  x <- x[!is.na(x)]
  if (length(x) == 0) return(NA_real_)
  if (require_all) return(as.numeric(all(x)))
  as.numeric(mean(x) >= threshold)
}

calc_strict_score <- function(df, require_all = FALSE, threshold = 0.5) {
  out <- df

  out$s_lipid <- apply(out[c("tg_dec","chol_dec")], 1, function(z)
    domain_score_prop(as.logical(z), threshold, require_all))
  out$s_inflam <- apply(out[c("crp_dec","pct_dec","il6_dec","tnfa_dec")], 1, function(z)
    domain_score_prop(as.logical(z), threshold, require_all))
  out$s_pancreas <- apply(out[c("amy_dec")], 1, function(z)
    domain_score_prop(as.logical(z), threshold, require_all))
  out$s_renal <- apply(out[c("cr_dec","bun_dec")], 1, function(z)
    domain_score_prop(as.logical(z), threshold, require_all))
  out$s_liver <- apply(out[c("bili_dec","alt_dec")], 1, function(z)
    domain_score_prop(as.logical(z), threshold, require_all))
  out$s_cbc <- apply(out[c("wbc_dec","neut_dec","pt_dec")], 1, function(z)
    domain_score_prop(as.logical(z), threshold, require_all))

  ss <- c("s_lipid","s_inflam","s_pancreas","s_renal","s_liver","s_cbc")
  complete <- rowSums(!is.na(out[ss])) == 6
  score <- ifelse(complete, rowSums(out[ss]), NA_real_)
  score
}

primary$score_50pct <- calc_strict_score(primary, threshold = 0.50, require_all = FALSE)
primary$score_all   <- calc_strict_score(primary, require_all = TRUE)

score_sensitivity_summary <- tibble(
  definition = c("Primary: >=1 available marker",
                 ">=50% of available markers",
                 "All available markers"),
  mean_score = c(mean(primary$score_primary, na.rm = TRUE),
                 mean(primary$score_50pct, na.rm = TRUE),
                 mean(primary$score_all, na.rm = TRUE)),
  sd_score = c(sd(primary$score_primary, na.rm = TRUE),
               sd(primary$score_50pct, na.rm = TRUE),
               sd(primary$score_all, na.rm = TRUE)),
  n_ge3 = c(sum(primary$score_primary >= 3, na.rm = TRUE),
            sum(primary$score_50pct >= 3, na.rm = TRUE),
            sum(primary$score_all >= 3, na.rm = TRUE)),
  n_ge4 = c(sum(primary$score_primary >= 4, na.rm = TRUE),
            sum(primary$score_50pct >= 4, na.rm = TRUE),
            sum(primary$score_all >= 4, na.rm = TRUE)),
  n_ge5 = c(sum(primary$score_primary >= 5, na.rm = TRUE),
            sum(primary$score_50pct >= 5, na.rm = TRUE),
```

```
            sum(primary$score_all >= 5, na.rm = TRUE))
)

score_sensitivity_summary
```

10. Rule-based laboratory-change pattern stratification

```
# The manuscript specifies a rule-based stratification, not unsupervised clustering.
# Pattern assignment uses the patient-level median ABSOLUTE relative change.
# Heatmap visualization may retain the signed relative change.

pattern_vars <- c(
  "tg_pct","cholesterol_pct","il6_pct","crp_pct","pct_pct",
  "amylase_pct","creatinine_pct","bun_pct","alt_pct",
  "bilirubin_pct","wbc_pct","pt_pct"
)

primary$n_pattern_available <- rowSums(!is.na(primary[pattern_vars]))

primary$median_abs_relative_change <- apply(
  abs(as.matrix(primary[pattern_vars])),
  1,
  function(z) {
    if (sum(is.finite(z)) < 8) return(NA_real_)
    median(z[is.finite(z)])
  }
)

eligible_pattern <- primary %>%
  filter(!is.na(median_abs_relative_change))

# Tertile cut points
cuts <- quantile(
  eligible_pattern$median_abs_relative_change,
  probs = c(1/3, 2/3),
  na.rm = TRUE,
  type = 2
)

# Keep tied values at a boundary in the same category.
eligible_pattern <- eligible_pattern %>%
  mutate(
    change_pattern = case_when(
      median_abs_relative_change > cuts[2] ~ "Greater-change",
      median_abs_relative_change < cuts[1] ~ "Lower-change",
      TRUE ~ "Intermediate-change"
    )
  )

table(eligible_pattern$change_pattern)

# If final verified data produce n = 40, 67, and 34, report those values.
# Otherwise report the actual group sizes returned by the verified dataset.
```

11. Time-window sensitivity analysis (post-BP sampling <=24 h)

```
within24 <- primary %>% filter(post_bp_hours <= 24)

time_window_vars <- c(
  "tg_pct","amylase_pct","il6_pct","crp_pct",
  "pct_pct","creatinine_pct","wbc_pct"
)

time_window_summary <- purrr::map_dfr(time_window_vars, function(v) {
  tibble(
    indicator = v,
    overall_median_relative_change =
      median(primary[[v]], na.rm = TRUE),
    within24_median_relative_change =
      median(within24[[v]], na.rm = TRUE),
    n_within24 = sum(!is.na(within24[[v]]))
  )
```

```
})

time_window_summary
```

12. Included vs excluded comparison for potential selection bias

```
selection_dat <- dat %>%
  filter(included == 1 | excluded_missing_pair == 1) %>%
  mutate(
    analysis_group = if_else(
      included == 1, "Included", "Excluded_missing_unpaired"
    )
  )

# Continuous variable: age
t.test(age ~ analysis_group, data = selection_dat)

# Categorical baseline characteristics
chisq_or_fisher <- function(tab) {
  if (any(chisq.test(tab)$expected < 5)) fisher.test(tab) else chisq.test(tab)
}

chisq_or_fisher(table(selection_dat$sex, selection_dat$analysis_group))
chisq_or_fisher(table(selection_dat$htg_ap, selection_dat$analysis_group))
chisq_or_fisher(table(selection_dat$severe_ap, selection_dat$analysis_group))
chisq_or_fisher(table(selection_dat$icu, selection_dat$analysis_group))

selection_summary <- selection_dat %>%
  group_by(analysis_group) %>%
  summarise(
    n = n(),
    age_mean = mean(age, na.rm = TRUE),
    age_sd = sd(age, na.rm = TRUE),
    male_n = sum(sex == "Male", na.rm = TRUE),
    htg_ap_n = sum(htg_ap == 1, na.rm = TRUE),
    severe_ap_n = sum(severe_ap == 1, na.rm = TRUE),
    icu_n = sum(icu == 1, na.rm = TRUE),
    .groups = "drop"
  )

selection_summary
```

13. Figure generation

```
# Figure 2A: ranking of median relative changes
fig2a_dat <- paired_results %>%
  arrange(median_relative_change_pct) %>%
  mutate(indicator = factor(indicator, levels = indicator))

p2a <- ggplot(fig2a_dat,
              aes(x = median_relative_change_pct, y = indicator)) +
  geom_col() +
  labs(x = "Median relative change (%)", y = "Laboratory parameter") +
  theme_classic()

ggsave("Figure2A_relative_change_ranking.png", p2a,
       width = 7, height = 5, dpi = 600)

# Figure 3: Spearman correlation matrix
png("Figure3_correlation_matrix.png", width = 1800, height = 1600, res = 250)
pheatmap::pheatmap(
  corr_r,
  display_numbers = TRUE,
  number_format = "%.2f",
  cluster_rows = FALSE,
  cluster_cols = FALSE,
  main = "Correlation Matrix of Changes in Major Laboratory Parameters"
)
dev.off()

# Figure 4: descriptive heatmap by rule-based change pattern.
# Calculate group medians of SIGNED relative changes.
```

```
heatmap_vars <- pattern_vars

heatmap_group <- eligible_pattern %>%
  group_by(change_pattern) %>%
  summarise(
    across(all_of(heatmap_vars), ~ median(.x, na.rm = TRUE)),
    .groups = "drop"
  )

hm <- as.matrix(heatmap_group[, heatmap_vars])
rownames(hm) <- heatmap_group$change_pattern

png("Figure4_rule_based_change_patterns.png", width = 2400, height = 1200, res = 250)
pheatmap::pheatmap(
  hm,
  cluster_rows = FALSE,
  cluster_cols = FALSE,
  display_numbers = TRUE,
  number_format = "%.0f",
  angle_col = 45,
  main = "Exploratory rule-based laboratory-change patterns"
)
dev.off()
```

14. Export analysis outputs

```
# Export key analysis tables for manuscript verification.

write.csv(paired_results,
          "paired_analysis_results.csv", row.names = FALSE)
write.csv(table_summary,
          "laboratory_summary_tables.csv", row.names = FALSE)
write.csv(bp_subgroup_results,
          "bp_modality_subgroup_results.csv", row.names = FALSE)
write.csv(score_sensitivity_summary,
          "score_sensitivity_results.csv", row.names = FALSE)
write.csv(time_window_summary,
          "time_window_sensitivity_results.csv", row.names = FALSE)
write.csv(selection_summary,
          "selection_bias_comparison.csv", row.names = FALSE)

# Save analysis-ready object
saveRDS(primary, "AP_BP_primary_analysis_object.rds")

# Final reproducibility record
capture.output(sessionInfo(), file = "R_sessionInfo.txt")
```

15. Mapping to manuscript analyses

Paired t-tests / Wilcoxon signed-rank tests: Sections 4–5.

95% CIs for paired changes: Section 4.

Exploratory subgroup analyses: Section 6.

Spearman correlation analysis: Section 7.

Exploratory six-domain laboratory change score: Section 8.

Stricter score sensitivity definitions: Section 9.

Rule-based greater/intermediate/lower-change pattern stratification: Section 10.

Post-BP <=24 h sensitivity analysis: Section 11.

Included vs excluded baseline comparison: Section 12.

Figure generation: Section 13.

Note on reproducibility

The code is designed to reproduce the analytical workflow described in the manuscript when applied to the final verified de-identified patient-level dataset. Exact numerical outputs depend on the final data values and variable mapping. Any reported manuscript values should be generated from the verified dataset rather than manually entered into the code.
