# Supplementary material for: Multisystem laboratory changes before and after blood purification in acute pancreatitis: a real-world descriptive analysis of 141 cases
Source: Front Med (Lausanne). 2026 Sep 23;13:1850265. doi: 10.3389/fmed.2026.1850265 (PMC13642849; doi:10.3389/fmed.2026.1850265)
Supplement: Supplementary file 2 [file Table1.docx]

Supplementary Table S1. Comparison of included patients and patients excluded because of missing/unpaired laboratory data.

| **Baseline characteristic** | **Included (n=141)** | **Missing/unpaired excluded (n=65)** | **P value** |
| --- | --- | --- | --- |
| Age, years | 47.01 ± 14.53 | **49.20 ± 15.10** | **0.33** |
| Male, n (%) | 97 (68.79) | **43 (66.15)** | **0.706** |
| Hypertriglyceridemia-associated AP, n (%) | 96 (68.09) | **40 (61.54)** | **0.357** |
| Severe AP, n (%) | 72 (51.06) | **40 (61.54)** | **0.161** |
| ICU admission, n (%) | 65 (46.10) | **38 (58.46)** | **0.099** |

Supplementary Table S2. Sensitivity analysis restricted to post-BP measurements obtained within 24 h.

| Indicator | Overall cohort relative change (%) | ≤24 h subgroup relative change (%) |
| --- | --- | --- |
| Triglycerides | −86.23 | **−84.90** |
| IL-6 | −86.79 | **−83.80** |
| Serum amylase | −86.67 | **−84.70** |
| CRP | −59.42 | **−57.10** |
| PCT | −57.93 | **−55.40** |
| Creatinine | −44.97 | **−42.30** |
| WBC | −44.54 | **−42.00** |

Supplementary Table S3. Sensitivity analyses of the exploratory multisystem laboratory change score using alternative domain definitions

| **Definition** | **Mean score** | **≥3 domains, n (%)** | **≥4 domains, n (%)** | **≥5 domains, n (%)** |
| --- | --- | --- | --- | --- |
| Primary: ≥1 available marker | 4.24±1.34 | 124 (87.94) | 100 (70.92) | 67 (47.52) |
| ≥50% of available markers* | 3.71±1.42 | 110 (78.01) | 82 (58.16) | 48 (34.04) |
| All available markers* | 3.18±1.39 | 91 (64.54) | 61 (43.26) | 30 (21.28) |
